# Comparative evaluation of ropivacaine and lidocaine as dental pulp anaesthesia

**DOI:** 10.6026/97320630017229

**Published:** 2021-01-31

**Authors:** Vijayapriyangha Senthilkumar, Sindhu Ramesh

**Affiliations:** 1 Postgraduate Student, Department of Conservative Dentistry and Endodontics Saveetha Dental College, Saveetha Institute Of Medical And Technical Science Chennai, India

**Keywords:** Inferior alveolar nerve block, Local anesthesia, Pulpal anesthesia, Ropivacaine, Symptomatic Irreversible pulpitis

## Abstract

Root canal therapy linked to pulpal diseases or trauma is common in modern dental care. The 2% Lidocaine which is considered as the gold standard has some drawbacks in pulpal anaesthesia. Ropivacaine has beneficial anaesthetic effects on pulpal anaesthesia. Therefore, it is of interest to compare and evaluate the pulpal aesthetic effect using 0.5% Ropivacaine and 2% Lidocaine in symptomatic irreversible pulpitis. A double blinded randomized controlled clinical trial consisting of 110 lower molar and premolar tooth with irreversible pulpitis cases for root canal therapy were selected and randomly divided into 2 groups. Group A: 2% lidocaine with epinephrine and Group B: 0.5% ropivacaine. The pulp sensibility tests with heat test, cold test and electric pulp test were completed. The preoperative pain score was measured with Visual Analogue Scale (VAS) pain scale. The classical inferior alveolar nerve block (IANB) technique was administered to all patients by a single operator. Subjects were asked for lip numbness and presence or absence of lip numbness. Postoperative pain scores were recorded during access opening and on placing files in the canal. There is no statistical difference between the groups during pre operative conditions. The mean pain scores within group A and group B is recorded. The difference was found to be statistically significant with p value ≤ 0.05. Significant difference between the mean values after and before the treatment is observed. However, there is no statistical significance between the mean pain scores between the access and pulp. The 0.5% Ropivacaine and 2% Lidocaine with epinephrine does not have any significant difference during access opening. However, 0.5% Ropivacaine groups were effective while placing the file in the canal. Thus, 0.5% Ropivacaine showed better results even though it was not statistically significant for further consideration in this context.

## Background

Pulpal inflammation may or may not be associated with toothache, which will be recognized as acute and chronic pulpitis based on the duration of the condition. The localized increase in tissue fluid pressure associated with acute inflammation usually results in toothache. Clinically, it can be divided into reversible and irreversible pulpitis. The treatment for reversible pulpitis will be with pulp protecting agents and for irreversible pulpitis is preferably root canal treatment. Local anesthesia is an effective method of pain control since 1884, which is used to initiate root canal procedure. Lidocaine is a short acting (vasodilator), where 2% Lidocaine is commonly used in dentistry. To increase the depth and duration of anesthesia, epinephrine was added to Lidocaine. Nonetheless, in 1964 epinephrine containing local anesthetic solution was contraindicated in hyperthyroidism and significant cardiovascular diseases (American Society of Anesthesiologists physical status grade 3-4). As well, adding vasoconstrictor reduces the pH of the solution (acidic), rendering the injections uncomfortable to the patients. Hence, search for a long‑acting local anesthetic agent continues for effective pain control for treatment of irreversible pulpitis [1-6]. The inferior alveolar nerve block technique (IANB), which commonly has high failure, rates, particularly in patients with irreversible pulpitis [3,7]. Studies in patients with irreversible pulpitis have reported failure rates with IANB of 44-81% [8-10] due to activated nociceptors in an inflamed pulp, which leads to a decrease in pain threshold and decreased efficacy of the IANB [11]. Therefore, substantial ongoing research is directed at improving the success rate of IANB in patients with irreversible pulpitis, by exploring different injection techniques, anaesthetic solutions [8], supplemental infiltration techniques [12,13], premedication before performing the IANB [14,15] and addition of an adjunct for local anaesthetic formulations [16-18]. Ropivacaine hydrochloride is a relatively new local amide anaesthetic. It was used in 1992 for the first time in the Royal Hospital for Women in Sydney and introduced for clinical use in 1996 [19,20]. Ropivacaine is similar in structure to bupivacaine, but it has optically pure S (-) enantiomeric from the parent chiral molecule p -Ropivacaine. Ropivacaine belongs to the group of local anesthetics, the pipecoloxylidides and it has a propyl group on the piperidine nitrogen atom when compared to bupivacaine, which has a butyl group. The use of the s (-) enantiomer, Ropivacaine instead of the racemic, bupivacaine, gives a wider safety margin with the same anaesthetic efficacy [21-25]. Because of its favourable qualities such as low toxicity, long duration of action and selectiveness for nerve fibres responsible for pain transmission than motor function, Ropivacaine has so far been successfully used in surgery, gynaecology and obstetrics, but is not currently available for dentists [22, 26-28]. Ropivacaine has a biphasic vascular effect, which could be useful in dentistry. In low concentration (0.063- 0.5%), it shows vasoconstriction per se and vasodilation at high concentration (1%) [29-31]. The maximum dose of 0.5% Ropivacaine for minor nerve blocks and infiltration is 200 mg. Only limited data are available concerning the dental use of Ropivacaine [32-34]. Therefore, it is of interest to document the comparative evaluation of 0.5% ropivacaine and lidocaine as dental pulp anaesthesia.

## Materials and Method:

### Study Design:

A prospective, single centre, Double Blinded Randomized Controlled Clinical Trial is designed.

### Ethical Approval:

Approval for the project was obtained from the Institutional Review Board and Ethical Board of Saveetha Institute of Medical and Technical Sciences, Chennai, India [SRB Reference No.SRB/SDMDS10/19/ENDO/01].

### Sample Size Determination:

Based on study done by E.Shadmehr et al, 2016, efficacy of Lidocaine with epinephrine or clonidine for inferior alveolar nerve block technique in patients with irreversible pulpitis and T.F.Krzeminski et al. 2011, efficacy of Ropivacaine and Articaine with epinephrine for infiltration anesthesia, sample size estimation was calculated using a nMaster software version 2.0 by applying the details in the sample size determination formula, where H0: P1 = P2 and H1: P1 ≠ P2. p1= proportion in the first group, p2=proportion in the second group, α= significance level and 1-b = power.

### Eligibility Criteria:

The samples were included in the study according to the following inclusion criteria: Subjects with the age group above 18 years, teeth with irreversible pulpitis, only lower posterior teeth (molars and premolars) and Pre operative pain score more than or equal to VAS score 4. And the subjects were excluded from the study if they have any of the following: Teeth with periapical lesions, periodontally compromised teeth, teeth with non- carious lesions and subjects suffering from any systemic conditions.

### Setting and Location:

The volunteer patients fitting the inclusion criteria described above were included in the study. The study participants for the study were recruited from the pool of patients in the Department of Conservative Dentistry and Endodontics at our Institution. The patients were informed about the procedure and informed consent was taken from all patients included in the study.

### Randomization:

Sequence Generation:

Randomization was done well in advance by a third person that was not related to the study. Randomization was done using block randomization procedure, assigned to Group A- 2% Lidocaine with epinephrine (n=55) to Group B- 0.5% Ropivacaine (n=55).

### Allocation Concealment:

SNOSE (sequentially numbered, opaque, sealed envelopes) method was implemented for allocation concealment, which concealed the sequence until interventions were assigned. A piece of paper containing randomized group number was sealed in the dark colored envelope containing respective serial number over it prepared by a third person. The group is mentioned clearly and sealed in the envelope. Patients were assigned their study numbers as they sequentially entered the study. The envelope was opened once the intervention was assigned. Based on the group assigned in the paper, respective treatment was carried out.

### Blinding:

Patients and the clinician were unaware of the type of treatment group and they were blinded. But the evaluator knew about the type of group. Hence, it is a double-blinded study. Patients were divided into two groups as Group A - 2% Lidocaine with epinephrine and Group B - 0.5% Ropivacaine.

### Treatment Procedure:

Prior to the treatment, a careful medical and dental history was taken. Preoperative data for each patient was recorded in the predesigned patient's chart, which includes age, sex and tooth number. The treatment and the study design were explained to the qualifying patients and informed consent was obtained from the voluntary patients who were willing to participate in the study. The confirmatory tests were done to the selected tooth by keeping the contra lateral tooth as control. Heat test, cold test and electric pulp testing (EPT) were done to confirm the diagnosis of symptomatic irreversible pulpitis. All selected teeth were randomly divided into two groups with 55 each (N=110) depending on the type of local anesthetic agent used. Randomization of the local anesthesia used was done by an envelope draw method for all the selected teeth, present either in different patients or in the same patient. Pre operative pain score was recorded for the selected tooth using visual analogue scale (VAS). Depending on the type of local anesthesia, the teeth were treated as follows:

### Group A:

2% Lidocaine with epinephrine (Neon Laboratories Ltd. Mumbai, India) loaded in a 26 gauge needle unlock syringe were administered by an inferior alveolar nerve block (IANB) on the quadrant of the selected tooth.

### Group B:

0.5% Ropivacaine (Neon Laboratories Ltd. Mumbai, India) loaded in a 26 gauge needle unlock syringe were administered by inferior alveolar nerve block (IANB) on the quadrant of selected tooth.

### Outcome Measures:

After administering the local anaesthesia, the presence or absence of lip numbness were noted and heat test, cold test and EPT test were done. After that the tooth was isolated with a rubber dam. Access opening of the tooth was done using endo access bur and Endo Z bur (Dentsply Sirona). The pain score during access opening was recorded using Visual analogue pain scale (VAS). After gaining access to the pulp chamber, the hand files such as K file of # 10 and 15 were placed in the canals in order to take working length and to clean and shape the canals. The pain score during placing the files were also noted using Visual analogue pain scale (VAS).

### Statistical Analysis:

Statistical analysis was performed using a statistical software program Windows, Version 21 (SPSS). The Normality tests Kolmogorov-Smirnov and Shapiro-Wilks tests results reveal that all variables follow Normal distribution. Therefore, a parametric test is applied to analyze the data. Data was entered in Microsoft excel spreadsheet and analyzed using SPSS software (version 21). For the test, a p value of < 0.05 is to be considered statistically significant. Chi Square test were used to assess the descriptive statistics, Independent t test were used to assess the mean difference between the treatment groups. One-way ANOVA were used to assess the significant differences in the pain scores within the treatment groups. Tukeys post hoc test were used to assess the multiple group comparison within the groups.

## Results:

A total of 110 teeth of patients were included in this clinical trial. Data analysis was done with the 110 teeth. Table 1 (see PDF) depicts the lip numbness present or absent after administering the anesthesia in both the groups. Table 2 (see PDF) depicts the pre-operative pain between the groups. The difference was not found to be significant statistically (p value≥0.05). Table 3 (see PDF) depicts the mean post-operative pain (access) between the groups. The difference was not found to be significant statistically (p value≥0.05). Table 4 (see PDF) depicts the mean post-operative pain (pulp) between the groups. The difference was not found to be significant statistically (p value≥0.05). Table 5 (see PDF) depicts the mean pain scores within group A and group B. The difference was found to be significant statistically (p value≤0.05). Table 6(see PDF) depicts multiple comparisons of mean pain scores preoperatively and postoperatively. There is a statistically significant difference between the mean values pre operative pain and postoperative pain after administering local anesthesia during access and when placing file (pulp). However, there is a no statistically significant difference between the mean values postoperative pain after administering local anesthesia during access and when placing file (pulp). Table 7 (see PDF) depicts multiple comparisons of mean pain scores preoperatively and postoperatively. There is a statistically significant difference between the mean values pre operative pain and postoperative pain after administering local anesthesia during access and when placing file (pulp) . However, there is a no statistically significant difference between the mean values postoperative pain after administering local anesthesia during access and when placing file (pulp).

##  Discussion:

This prospective double blinded randomized clinical trial compared the pulpal anesthetic effect using 2% Lidocaine with epinephrine and 0.5% Ropivacaine by inferior alveolar nerve block technique in patients with symptomatic irreversible pulpitis. The onset of anesthesia is an important issue for anesthesia success evaluation. The onset of anesthesia in inferior alveolar nerve block and infiltration injection are different and the later technique provides quicker anesthesia [35]. The pulpal status and the diagnosis of pulp along with periapical disease at the time of procedure may be important issues in the success rate of anesthesia [35]. Potocnik et al. 2006, studied the in vitro effects of Lidocaine and articaine, both at concentrations of 2% and 4%, in addition to 3% mepivacaine, on decreases in the amplitude of the action potential produced by sensory nerve fibers in rats following supramaximal electrical stimulation. These authors reported that for all tested anesthetic solutions, there was complete disappearance of the action potential produced by the C-fibers but not the A-fibers. The VAS is the most widely used tool for estimating both severities of pain and to judge the extent of pain relief [36, 37]. The VAS is a continuous scale, which is comprised of a horizontal (HVAS) or vertical (VVAS) line, usually 100 mm long, anchored by two verbal descriptors (i.e., "no pain" and "worst imaginable pain") [36, 38,39]. Patients were asked to rate the "current" pain intensity or pain intensity "in the last 24 h". The VAS is a simple instrument that does not warrant using a sophisticated device. It is highly sensitive in detecting the effects of treatment, and its results can be analyzed by parametric tests [40]. Minimal translation difficulties have led to an unknown number of cross-cultural adaptations [39]. Despite, this tool is suitable for use with older children and adults. The requirement for a marking and for being able to visualize and mark the line makes the VAS unfeasible to use in the emergency situation. On the other hand, most experts believe that the VAS offers little practical advantage over verbal reports in the clinical practice [36,39]. In root canal therapy, pain control is very crucial, and the most important way to prevent pain is using local anesthetic drugs. These drugs have a peripheral effect and block the transmission of nerve impulses. The long-acting anesthetic drugs can provide pain control for 6 hours or longer. Factors affecting the anesthetic drug efficacy are the type of the applied drug, amount, proper injection site, injection velocity and dosage of the injected drug. The presence of inflammation at the site of injection is another important aspect, which should not be overlooked. According to clinical experiences, the teeth with pulpal involvement (pulpitis) do not anesthetize completely. Success rate of inferior alveolar nerve block for teeth without any sign of inflammation is reported about 85-90% [41,42] whereas, success rate for teeth with inflammation is reported <20% or very poor [41,43,44]. There are different types of voltage-dependent gates. One type of these gates, which is called tetrodotoxin-resistant, exists in the sensory nerve fiber, which might increase in number in the inflammatory situation. Unlike other voltage- dependent gates, this gate is hardly blocked by Lidocaine [45], which might explain why teeth with irreversible pulpitis do not anesthetize easily. Numbers of local anesthetic solutions are introduced to overcome the anesthetic resistance of teeth with irreversible pulpitis. One such is Ropivacaine, a long acting local anesthesia. The onset of 2% Lidocaine with 1:200,000 epinephrine came out to be much faster as compared to 0.75% Ropivacaine. This was in concurrence with [33,46]. This delay in onset of action may be attributed to the highest pKa value of Ropivacaine, intermediate lipid solubility of Ropivacaine, and complexity of injection [33,47]. However, El‑Sharrawy and Yagiela et al showed rapid onset of action with 0.75% and 0.5% of Ropivacaine for inferior alveolar nerve block [48]. Though studies say, there is a high failure rate of IANB and insufficient pain management in patients with irreversible pulpitis [8] has led to the use of adjuvants to increase the success rate of anaesthesia. Previous well-designed trial studies investigated adjuvants such as meperidine, diphenhydramine, hyaluronidase and sodium bicarbonate to increase IANB success rates. However, the studies reported that none of the solutions were able to improve anaesthetic success of the IANB significantly better than Lidocaine and epinephrine [42,49, 50]. Lip numbness has been used as an indicator of a clinically successful block, but it does not guarantee for successful pulpal anaesthesia. The success rate of anesthesia was defined as the ability to penetrate into dentine, entering the pulp and instrumentation (advance instruments into the coronal part of the canal pulp) without pain (VAS score of zero) or mild pain (VAS rating ≤54 mm). Lidocaine with epinephrine solution exhibited a 29% anaesthesia success rate for IANB in patients with irreversible pulpitis [51]. The success was similar to that reported in previous studies of endodontic patients with irreversible pulpitis, ranging from 19% to 56% [16,52, 53]. The studies showed that 0.5% Ropivacaine with epinephrine are equivalent to bupivacaine and effective than articaine [32,54]. But in both the studies of Ropivacaine in relation to pulpal anesthesia, they have checked only lip numbness, time onset of action and efficacy with electric pulp testing. In this present study, 0.5% Ropivacaine was compared to 2% Lidocaine with epinephrine, where the lip numbness present in group A were about 90.9% and in group B were about 94.5%. The preoperative pain between two groups was not statistically significant (p>0.05). Post operative pain during access opening and pain on keeping file in pulp were not statistically significant between two groups (p>0.05). Under the limitations of this clinical trial, it is concluded that there is a significant difference in post operative pain between two groups 2% Lidocaine with epinephrine and 0.5% Ropivacaine. And there is a significant difference within the groups (p<0.05).

## Conclusion:

We show that the 2% Lidocaine with epinephrine group had 90.9 % of lip numbness and 0.5% Ropivacaine had 94.5% of lip numbness after anesthesia. The 0.5% Ropivacaine and 2% Lidocaine with epinephrine does not have any significant difference during access opening. However, 0.5% Ropivacaine was effective during access opening. The 0.5% Ropivacaine and 2% Lidocaine with epinephrine does not have any significant difference while placing file in the canal. However, 0.5% Ropivacaine groups were effective while placing files in the canal. The 0.5% Ropivacaine showed better results even though it is not statistically significant. Hence, it can also be used as a local anesthesia in cases with symptomatic irreversible pulpitis.

## Figures and Tables

**Figure 1:**
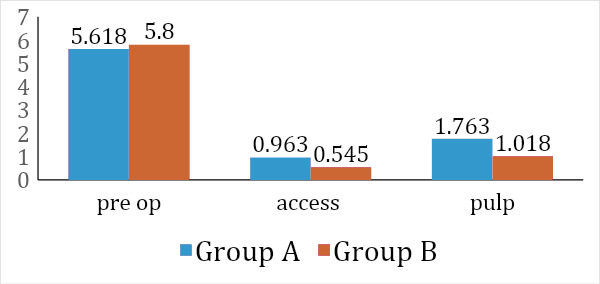
: Comparison of mean pain scores within the treatment groups.
